# Interferon gamma protects neonatal neural stem/progenitor cells during measles virus infection of the brain

**DOI:** 10.1186/s12974-016-0571-1

**Published:** 2016-05-13

**Authors:** Kristen N. Fantetti, Erica L. Gray, Priya Ganesan, Apurva Kulkarni, Lauren A. O’Donnell

**Affiliations:** Division of Pharmaceutical Sciences, Mylan School of Pharmacy, Duquesne University, 600 Forbes Ave, Pittsburgh, PA 15282 USA

**Keywords:** Anti-viral, Glia, Immune response, Interferon-γ, Measles virus, Neonate, Neurogenesis, Neural stem cell, STAT signaling

## Abstract

**Background:**

In the developing brain, self-renewing neural stem/progenitor cells (NSPC) give rise to neuronal and glial lineages. NSPC survival and differentiation can be altered by neurotropic viruses and by the anti-viral immune response. Several neurotropic viruses specifically target and infect NSPCs, in addition to inducing neuronal loss, which makes it difficult to distinguish between effects on NSPCs that are due to direct viral infection or due to the anti-viral immune response.

**Methods:**

We have investigated the impact of anti-viral immunity on NSPCs in measles virus (MV)-infected neonates. A neuron-restricted viral infection model was used, where NSPCs remain uninfected. Thus, an anti-viral immune response was induced without the confounding issue of NSPC infection. Two-transgenic mouse lines were used: CD46+ mice express the human isoform of CD46, the MV entry receptor, under the control of the neuron-specific enolase promoter; CD46+/IFNγ-KO mice lack the key anti-viral cytokine IFNγ. Multi-color flow cytometry and Western Blot analysis were used to quantify effects on NSPC, neuronal, and glial cell number, and quantify effects on IFNγ-mediated signaling and cell markers, respectively.

**Results:**

Flow cytometric analysis revealed that NSPCs were reduced in CD46+/IFNγ-KO mice at 3, 7, and 10 days post-infection (dpi), but were unaffected in CD46+ mice. Early neurons showed the greatest cell loss at 7 dpi in both genotypes, with no effect on mature neurons and glial cells. Thus, IFNγ protected against NSPC loss, but did not protect young neurons. Western Blot analyses on hippocampal explants showed reduced nestin expression in the absence of IFNγ, and reduced doublecortin and βIII-tubulin in both genotypes. Phosphorylation of STAT1 and STAT2 occurred independently of IFNγ in the hippocampus, albeit with distinct regulation of activation.

**Conclusions:**

This is the first study to demonstrate bystander effects of anti-viral immunity on NSPC function. Our results show IFNγ protects the NSPC population during a neonatal viral CNS infection. Significant loss of NSPCs in CD46+/IFNγ-KO neonates suggests that the adaptive immune response is detrimental to NSPCs in the absence of IFNγ. These results reveal the importance and contribution of the anti-viral immune response to neuropathology and may be relevant to other neuroinflammatory conditions.

**Electronic supplementary material:**

The online version of this article (doi:10.1186/s12974-016-0571-1) contains supplementary material, which is available to authorized users.

## Background

Central nervous system (CNS) viral infections during fetal and postnatal periods significantly alter neurodevelopment, leading to long-term cognitive deficits, blindness, hearing loss, and neurological disease [1–4]. Neurotropic viruses cause CNS damage by directly infecting and killing resident CNS cells (polio virus, Semliki Forest virus) and/or by inducing an immune response that causes inflammation and encephalitis (measles virus, human immunodeficiency virus, cytomegalovirus, herpes simplex virus, West Nile virus) [5–10]. Ideally, the anti-viral immune response must carefully control a viral infection with minimal damage to non-renewable neurons. An excessive or prolonged inflammatory response can lead to bystander-induced damage and can contribute to neuropathology [11, 12].

In the developing CNS, self-renewing neural stem precursor cells (NSPC) provide a pool of progenitor cells that can mature into functional neurons or glia [13]. Neuronal loss is a common pathological outcome of CNS infections, but effects on NSPC function may also contribute to viral-induced neuronal loss. Neurotropic viruses can disrupt the survival, proliferation, and maturation of NSPCs, which ultimately impairs neurogenesis. Lack of CNS repair may be caused by a deficit in the NSPCs pool, disrupted formation of new neurons from NSPCs, or changes in cell fate. Furthermore, some viruses specifically target NSPCs [14, 15]. Many studies that measured NSPC survival and differentiation during viral infections have done so with viruses that infect and replicate in NSPCs [16–18]. This makes it experimentally difficult to distinguish between which component contributes to neuropathology: primary viral infection or bystander inflammation.

The immune response in the CNS can non-cytolytically control viral replication and spread via anti-viral cytokines. IFNγ is a pro-inflammatory cytokine produced by activated natural killer (NK) cells, natural killer T-cells (NKT), and T-cells and are required for non-cytolytic control of measles virus [19–21] and other neurotropic viruses [22–26]. During the anti-viral immune response, the release of pro- and anti- inflammatory cytokines also influences NSPC survival and neurogenesis [27, 28]. There is conflicting evidence as to whether IFNγ is pro-neurogenic [29–32] or anti-neurogenic [33–37], depending on the age studied (neonate or adult), model system, and the presence or absence of the inflammatory response. Moreover, there is conflicting evidence as to whether IFNγ induces apoptosis in neural cells, as human oligodendrocyte precursors are killed by IFNγ, but primary neurons are protected from other neurotoxic insults by IFNγ signaling [38, 39].

We hypothesized that IFNγ protects neonatal NSPCs during a CNS viral infection. To evaluate the effects of the anti-viral immune response on NSPCs and determine if loss of IFNγ alters these effects, a transgenic mouse model of neuron-restricted measles virus (MV) infection was used. NSE-CD46+ mice express the human isoform of CD46, a receptor by which MV binds and enters cells. In this model, CD46 expression is driven by the neuron-specific enolase (NSE) promoter, restricting human CD46 expression and MV infection to mature CNS neurons [40]. In order to study the role of IFNγ, we also use CD46+ mice that have been backcrossed to IFNγ-KO mice (CD46+/IFNγ-KO). Thus, modulations in NSPC function during MV infection can be examined with or without the contribution of IFNγ. Importantly, NSE is not expressed by NSPCs that express nestin [41], which spares NSPCs from MV infection in our model. Therefore, we are able to study the impact of the anti-viral immune response on NSPCs, without the confounding issue of direct viral infection of these cells.

Our study focuses on the fate of NSPCs that remain uninfected during a neonatal viral infection. Using the NSE-CD46+ model, we evaluated the bystander effects of the anti-viral immune response on NSPC function. Multi-color flow cytometry was used to quantify effects on the number of NSPCs, on cells specified to the neuronal lineage, and on cells specified to the glial lineage in vivo. We observed significant NSPC loss in MV-infected CD46+/IFNγ-KO pups at 7 and 10 dpi, but not in the infected CD46+ pups, suggesting that IFNγ protects NSPCs during a neuroinflammatory response. However, early neuronal cells were not protected; MV-infected CD46+ and CD46+/IFNγ-KO pups both displayed loss of early neurons and a decrease in neurogenesis. Furthermore, mature neurons and cells specified to the glial lineage were unaffected during the course of infection. Signal transducers and activators of transcription-1 (STAT1), a key downstream signal transducer for IFNγ, was phosphorylated in hippocampal explants from both CD46+ and CD46+/IFNγ-KO pups, suggesting that other cytokines in the inflammatory response were contributing to STAT activity in the absence of IFNγ. These results are the first to indicate that IFNγ protects NSPCs during a neonatal viral infection and furthers our understanding of what cell types are susceptible to the effects of the anti-viral immune response.

## Methods

### Animals

NSE-CD46+ and NSE-CD46+/IFNγ-KO transgenic mice were provided by Glenn Rall (Fox Chase Cancer Center) [19, 40]. Animal use protocols (#1408-10) were reviewed and approved by the Duquesne University Animal Care and Use Committee.

### Measles virus and BrdU injections

MV-Edmonston was purchased from American Type Culture Collection (ATCC) and passaged and titred in Vero cells. On postnatal day 2 (P2), pups were intracerebrally injected with measles virus (10^4^ PFU in a 10 μl volume) using a 1 cc syringe (BD Biosciences) and 27^1/2^G needle (BD Biosciences). Control pups were injected with an equal volume of media from mock-infected Vero cells. Twenty-four hours prior to being sacrificed on the indicated day post-infection (dpi), control and MV-infected mice were injected intraperitoneally with 50 μg/g 5-bromo-2′-deoxyuridine (BrdU; BD Biosciences), using a 1-cc syringe and 30G x ½-in needle (BD Biosciences).

### Dissociation of whole-brain tissue and flow cytometric analysis

Mice were anesthetized with isoflurane, and entire brains were removed from control and MV-infected neonates on 3, 7, and 10 dpi. Whole-brain tissue was processed into single-cell isolates and labeled as described [17, 42], with modifications. Briefly, brain samples were dissociated into single-cell suspensions following incubation with TrypLE Select (Life Technologies)/200 U/ml DNaseI (Roche)/1 mM MgCl_2_ (Life Technologies). Cell suspensions were filtered through 70 μm and 40 μm cell strainers (Fisher Scientific) and counted by trypan blue exclusion. Further, 10^6^ cells were labeled using the FITC BrdU Flow Kit (BD Biosciences) and the following antibodies: APC-A2B5 (1:10; Miltenyi Biotec), PE-CD24 (1:50; BD Biosciences), FITC BrdU (1:50; BD Biosciences), PE-Nestin (1:10; R&D Systems), FITC-NeuN (1:50; Millipore), rabbit anti-doublecortin (DCX, 1:50; Santa Cruz Biotechnology Inc) and secondary fluorescent-conjugated goat anti-rabbit IgG-PE (1:100; eBioscience), rabbit anti-GFAP (1:250; Dako), and goat anti-rabbit IgG-PE (1:100; eBioscience) secondary. Labeling with cell surface markers (A2B5, CD24) was performed before fixation and permeabilization.

Cells were analyzed the same day on a BD Accuri™ C6 flow cytometer (BD Biosciences). For each brain sample, 250,000 events were run for analysis. Dead cells and debris were excluded based on 7-AAD (BD Biosciences) staining as described [43, 44] (Additional file 1: Figure S1). Positively and negatively labeled cell populations and gating parameters were based on unlabeled and isotype control-labeled cells.

### Immunohistochemical analysis of brain tissue

Control and MV-infected mice were anesthetized with isoflurane and perfused with 4 % paraformaldehyde/PBS. Fixed brains were sliced along the midline and cryoprotected in a 30 % sucrose/PBS solution at 4 °C. Brains were immersed in tissue embedding compound (Fisher) and frozen in a dry ice/isopentane bath. Sagittal cryosections (16 μM) were cut on the cryostat (Microm HM550, Thermo Scientific) and stored at −80 °C until staining. Antigen retrieval was performed by heating the tissue for 30 min at 80 °C in a 10-mM sodium citrate solution. Standard immunohistochemistry was performed to detect MV-infected cells (mouse anti-matrix protein and mouse anti-hemagglutinin, 1:400; Millipore), neural stem/progenitor cells (chicken anti-nestin for co-labeling with MV (1:200; Novus Biologicals) or mouse anti-nestin for co-labeling with NeuN (1:400; Millipore)), and mature neurons (NeuN (guinea pig anti-NeuN) 1:500; Millipore). Sections were incubated with the primary antibody overnight at 4 °C in 4 % goat serum/PBS. Then, the slides were washed ×3 with PBS and incubated with the secondary antibody (goat anti-guinea pig-488, donkey anti-mouse-555, and goat anti-chicken-488; all at 1:000 from Thermo-Fisher) and Hoechst33342 stain (Molecular Probes) for 1 h at 25 °C. For all histological analyses, at least three sagittal sections per brain were examined, and at least four mice per experimental group were assessed.

### Apoptosis in MV-infected brain tissue

Brains from control and MV-infected mice (7 dpi) were collected following the procedure for the immunofluorescence assay. Sagittal brain sections were fixed in 3.7 % formaldehyde and subjected to terminal deoxynucleotidyl transferase dUTP nick end labeling (TUNEL; (TdT-FragEL DNA fragmentation detection kit; Millipore) using diaminobenzidine as a substrate. For counting of TUNEL-positive cells, four non-overlapping fields (×20) were chosen by a blinded examiner moving dorsally to ventrally across the slice. TUNEL-positive cells were counted on an Olympus BX41 Laboratory Microscope (Olympus Corporation). Three sagittal slices were counted from each brain, with three mice assayed per condition. The average number of TUNEL-positive cells per ×20 field ± standard error was determined.

### Western Blot analysis on hippocampal explants

Samples were processed and analyzed as described [45, 46], with modifications. For samples used for analysis of phosphorylated proteins, pups were perfused with 10 mM sodium fluoride in ice cold ×1 PBS prior to brain dissection in order to inactivate phosphatases [47]. The right and left hippocampus were dissected from each animal [48] and explants were combined, weighed, and sonicated in ×1 cell lysis buffer (Cell Signaling Technologies) supplemented with Protease Inhibitor Cocktail (Sigma). Samples were sonicated for 5–10 pulses for 1–2 s each (Misonix Inc. Model XL2020). The total protein content of cell lysates was quantified using the Pierce® BCA Protein Assay Kit (ThermoScientific) and the 1420 Multilabel Counter Victor3 plate reader (Perkin Elmer). Twenty micrograms of total protein was loaded for all proteins but STAT2 and STAT2-P (40 μg was loaded). Proteins were separated on NuPAGE 7 % Tris-Acetate (Nestin, STAT1, STAT1-P, STAT2, STAT2-P, STAT3, STAT3-P; Invitrogen) or NuPAGE 10 % Bis-Tris (GFAP, DCX, βIII-tubulin; Invitrogen) gels and semi-dry transferred (BioRad TransBlot Semi-Transfer Cell) to immobilon-FL membranes (Millipore). Membranes were incubated in 50 % odyssey blocking buffer (LI-COR Biosciences) in 0.1 % PBS/Tween (PBS/Tw; Sigma) for 30 min at room temperature, washed in PBS/Tw and incubated in the following primary antibodies, diluted in blocking solution, overnight at 4 °C on a shaker: mouse anti-nestin (1:250; Millipore), rabbit anti-DCX (1:1,000; Abcam), rabbit anti-βIII-tubulin (1:1,000; Cell Signaling Technologies), rabbit anti-GFAP (1:1,000; Dako), rabbit anti-STAT1 (1:1,000; BD Biosciences), mouse anti-STAT1-P (1:1,000; BD Biosciences), rabbit anti-STAT2 (1:1,000; Millipore), rabbit anti-STAT2-P (1:500; Millipore), mouse anti-STAT3 (1:2500; BD Biosciences), rabbit anti-STAT3-P (1:1,000; Cell Signaling Technologies), mouse anti-GAPDH (1:10,000; Millipore), and rabbit anti-GAPDH (1:3,000; Cell Signaling Technologies). Membranes were washed three times in PBS/Tw, incubated with secondary antibodies (infrared 800 or 700 nm anti-rabbit or anti-mouse 1:10,000; LI-COR Biosciences) diluted in blocking solution, for 1 h at room temperature and washed again in PBS/Tw. Membranes were imaged and quantified with the Odyssey® Infrared Imager (LI-COR Biosciences). Protein levels were normalized to GAPDH as a protein loading control.

### Statistical Analysis

Data are presented as the mean ± standard error (SE). Means represent data from a minimum of three independent experiments of 3–6 neonates for flow cytometry experiments and a minimum of four hippocampal explants for Western Blot experiments. Comparisons between control and MV-infected experimental groups in flow cytometry experiments were performed by three-way ANOVA followed by a Tukey’s multiple-comparison test, using SPSS statistics (IBM). Comparisons made in TUNEL assays and Western Blot experiments were performed by two-way ANOVA followed by a Tukey’s multiple-comparison test using GraphPad Software (GraphPad, Inc.). A *p* value of less than 0.05 was considered statistically significant.

## Results

### IFNγ protects neural stem/progenitor cells (NSPCs), but not early neurons, during viral infection of the neonatal brain

**Fig. 1:**
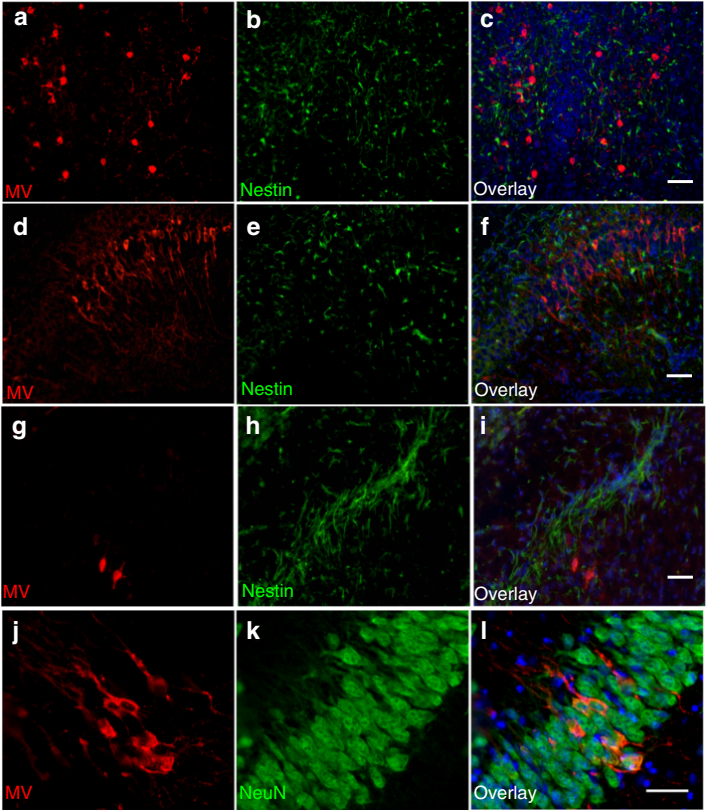
MV infects neurons, but not NSPCs, in CD46+ mice. Sagittal brain sections from MV-infected CD46+ mice were collected at 10 days post-infection (dpi) and stained for MV (*red*) and nestin (*green*; **a**–**i**) or MV (*red*) and NeuN (*green*; **j**–**l**). All sections were stained with Hoechst (*blue*) to visualize nuclei. Representative images are shown from different brain regions, including the thalamus (**a**–**c**), CA1 region of the hippocampus (**d**–**f**), fiber tracts of the medial forebrain bundle system (**g**–**i**), and the dentate gyrus (**j**–**l**). MV staining was restricted to NeuN+ cells (**j**–**l**). Co-localization with nestin and MV was not observed in any brain region (**a**–**i**). *Panels* **a**–**i**: Magnification = ×20; *scale bar* = 50 μm. Panels **j**–**l**: Magnification = ×40; *scale bar* = 25 μm

We first confirmed that MV infection is limited to CNS neurons in CD46+ neonates. Previous studies have demonstrated that MV antigen co-localizes with neuronal markers, but co-localization with markers for NSPCs (nestin) has not been investigated previously. MV+ cells were noted in the thalamus, hippocampus, and cerebellum early in infection (3 days post-infection (dpi); data not shown), with subsequent MV spread in the cerebral cortex at other time points (7–10 dpi, Fig. 1). Nestin+ cells were found in the vicinity of MV+ cells in multiple brain regions (Fig. 1, a–i); however, nestin and MV staining did not co-localize in any cells. Markers for mature neurons (NeuN, J-L) showed nuclear staining of MV+ cells, demonstrating that MV infection is limited to mature neurons.

We investigated whether a lack of IFNγ might impact the proportion of NSPCs or cells specified to the neuronal lineage during the course of infection. CD46+ and CD46+/IFNγ-KO pups were mock or MV-infected on postnatal day 2 (P2) and whole brains were harvested at 3, 7, or 10 days post-infection (dpi). The study endpoints reflect different phases of disease progression and correspond to when the specific arms of the immune system are active in the CD46+ model in both adults and neonates (unpublished observations and [49]). Three dpi corresponds to the initial phase of infection when the innate immune response predominates, including infiltration of IFNγ-producing natural killer (NK) cells. This is also the first day of detectable MV antigen and T-cell infiltration in the CNS [49]. At 7 dpi, the adaptive immune response predominates with peak infiltration of IFNγ-producing T-cells [49, 50]. Peak T-cell infiltration continues until 10 dpi, when severe signs of clinical disease and death occur in neonates.

**Fig. 2:**
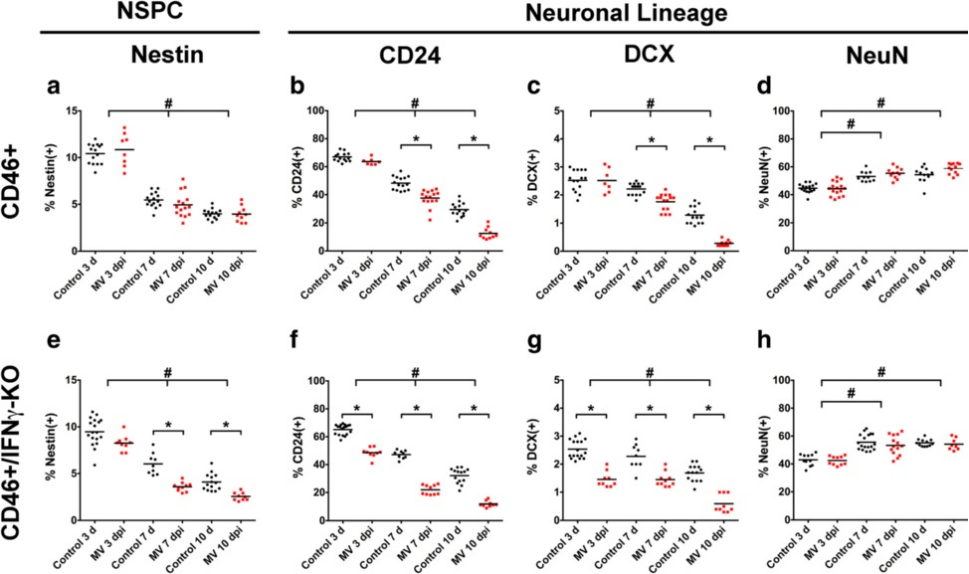
IFNγ preserves the NSPC population, but not early neurons. CD46+ (**a**–**d**) and CD46+/IFNγ-KO (**e**–**h**) control and MV-infected neonatal whole-brain isolates labeled for multipotent NSPCs (nestin: **a**, **e**) and cells specified to the neuronal lineage (CD24: **b**, **f**; DCX: **c**, **g**; NeuN: **d**, **h**), at 3, 7, and 10 dpi. The percentage of nestin + cells was significantly reduced at 7 and 10 dpi in MV-infected IFNγ-KO (**e**), but not MV-infected CD46+ mice (**a**). Significant CD24+ (**b**, **f**) and DCX+ (**c**, **g**) immature neuron cell loss occurred in both genotypes. Mature NeuN+ cell number was unaffected (**d**, **h**). *p < 0.01 significantly different uninfected versus MV-infected; #*p* < 0.05 significantly different between dpi

Cell isolates from whole brains were labeled with markers for multipotent neural stem/progenitor cells (nestin), immature neurons (CD24, doublecortin (DCX)), and mature neurons (NeuN), and multi-color flow cytometry was used to quantify the percentage of positively labeled cells. We observed an age-dependent decrease in the percentage of NSPCs (A, E) and early neurons (B, C, F, G), and an age-dependent increase in NeuN+ mature neurons (D, H) in both CD46+ and CD46+/IFNγ-KO genotypes (Fig. 2). Therefore, the percentage of positively labeled cells was compared between control and MV-infected brains at each dpi within each mouse genotype.

Overall, we observed significantly greater NSPC and immature neuron cell loss in MV-infected pups that lack IFNγ (CD46+/IFNγ-KO; Fig. 2e–g) compared to CD46+ pups (Fig. 2a–c) by three-way ANOVA (*p* < 0.001). The percentage of nestin + cells was reduced at 3 dpi, the first day of detectable MV antigen and T-cell infiltration in the CNS, and was significantly decreased at 7 and 10 dpi (Fig. 2e) in CD46+/IFNγ-KO pups, when the adaptive immune response and T-cell infiltration peak [49]. In contrast, nestin+ cells were maintained at all dpi in CD46+ mice in comparison to mock-infected mice (Fig. 2a), suggesting that IFNγ preserves this population during a CNS viral infection.

CD24+ and DCX+ immature neurons were most vulnerable to cell loss in CD46+ MV-infected brains at 7 and 10 dpi (Fig. 2b, c), but were lost at all dpi in MV-infected IFNγ-KO mice (Fig. 2f, g). Furthermore, the reduction in the percentage of CD24+ and DCX+ cells at 7 dpi was nearly twofold greater in CD46+/IFNγ-KO pups compared to CD46+ pups. When the percent change in positively labeled cells was compared between control and MV-infected animals at 7 dpi, IFNγ-KO mice displayed a 53 and 37 % decrease in CD24+ and DCX+ cells, respectively (Fig. 2f, g), compared to CD46+ mice (CD24+ 22 %; DCX+ 20 %; Fig. 2b, c). Conversely, there was no significant difference in the percentage of NeuN+ mature neurons, between control and MV-infected brains at all dpi, in both genotypes (Fig. 2d, h). From these results, we suspect that the anti-viral immune response is detrimental to NSPCs and that IFNγ may be protective against the detrimental effects of the immune response for NSPCs during a neonatal viral infection.

### Loss of IFNγ does not alter glial differentiation

**Fig. 3:**
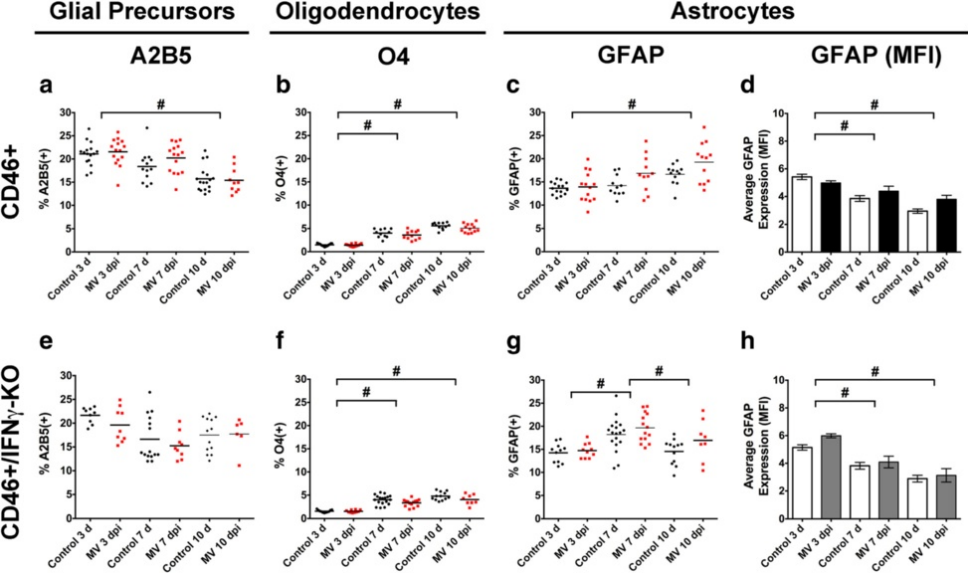
IFNγ does not alter glial differentiation. CD46+ (**a**–**d**) and CD46+/IFNγ-KO (**e**-**h**) control and MV-infected neonatal whole-brain isolates were labeled for early glial precursors (A2B5: **a**, **e**), oligodendrocytes (O4: **b**, **f**) and astrocytes (GFAP: **c**, **g**). The average GFAP mean fluorescent intensity (MFI) was quantified and normalized to the MFI of the isotype control (**d**, **h**). There was no significant difference between control and MV-infected pups for the glial cell populations in either genotype. A significant decrease in %A2B5+ cells was observed in CD46+ (**a**), but not IFNγ-KO mice (**d**) mice from 3 to 10 dpi and a significant increase in O4+ oligodendrocytes was observed from 3 to 10 dpi in both genotypes (**b**, **e**). Age-dependent differences in GFAP expression also occurred (**d**, **h**). *p < 0.01 significantly different uninfected versus MV-infected; #*p* < 0.05

CNS viral infections are often associated with increased glial activation and/or impaired glial function. Manchester et al. [50] reported widespread glial activation and astrocytic GFAP staining in MV-infected NSE-CD46+ pups at 7 dpi, leading us to speculate that the reduction in NSPC and neuronal cell number may be attributed to NSPCs being specified to the glial lineage and adopting a glial cell fate in MV-infected brains. Additionally, microglia and astrocytes are IFNγ-responsive, suggesting there may be IFNγ-dependent effects on glial function [11, 51]. We evaluated whether the anti-viral immune response and a lack of IFNγ impacted glial cell number. Cell isolates from mock- and MV-infected brains were labeled with A2B5, O4, or GFAP, and flow cytometry was used to quantify the percentage of glial precursors, oligodendrocytes, and astrocytes, respectively. Overall, glial cell number between control and MV-infected brains from CD46+ (Fig. 3a–c) and CD46+/IFNγ-KO (Fig. 3e–g) pups was unaffected at all dpi examined. Age-related differences in A2B5+ glial precursors (Fig. 3a) and O4+ oligodendrocytes (Fig. 3b, f) were observed in the presence and absence of the virus. Unexpectedly, we did not observe a significant effect on the percentage of GFAP+ cells (Fig. 3c, g) nor on the expression level of GFAP (Fig. 3d, h; MFI: Mean Fluorescent Intensity) between control and MV-infected pups in both types of mice, suggesting that there was minimal reactive gliosis in the whole brain. From these results, we concluded that the reduction in NSPC and early neuronal cell number was not due to NSPCs adopting a glial cell fate and next considered whether the loss of NSPCs and early neurons was due to effects on cell proliferation and/or aberrant neuronal differentiation.

### MV infection and loss of IFNγ do not alter BrdU uptake by nestin+ cells

IFNγ has been shown to either promote or inhibit NSPC proliferation and can regulate neurogenesis [30, 31, 33, 34, 36, 52–55]. One mechanism by which IFNγ may preserve the NSPC pool in CD46+ mice is by promoting NSPC proliferation. Similarly, the loss of early neurons may be attributed to either a lack of early neuron/neuroblast proliferation and/or impaired neuronal differentiation. We characterized the effects of MV infection and loss of IFNγ on the proliferation of nestin+, CD24+, and DCX+ cells, by isolating cells from pups that were injected with BrdU 24 h prior to harvest to label actively dividing cells. The proportion of BrdU+ cells within each cell population was quantified by flow cytometry.

**Fig. 4:**
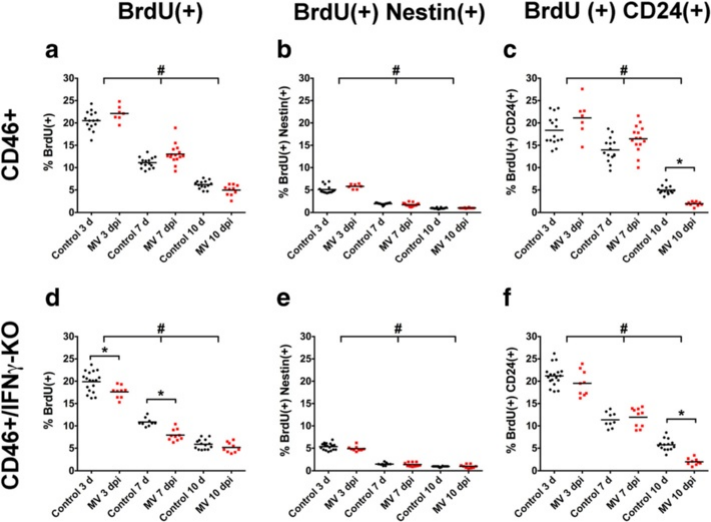
MV infection does not alter BrdU uptake by NSPCs. CD46+ (**a**-**c**) and CD46+/IFNγ-KO (**d**-**f**) pups were injected with BrdU 24 h before whole-brain isolates were processed and labeled for BrdU+ cells (**a**, **d**), or BrdU(+)Nestin(+) (**b**, **e**), and BrdU(+)CD24(+) (**c**, **f**) double-positive cells. Reduced BrdU uptake was observed in MV-infected CD46+/IFNγ-KO pups at 3 and 7 dpi (**d**), but not CD46+ mice (**a**). BrdU+ cells within the Nestin+ population showed no significant difference between control and MV-infected pups in both genotypes, but a significant reduction with age (**b**, **e**). The percentage of BrdU+ cells within the CD24+ cell population was reduced at 10 dpi (**c**, **f**), when severe illness and death occurs. *p < 0.01 significantly different uninfected versus MV-infected; #*p* < 0.05 significantly different between dpi

Reduced BrdU uptake was observed in MV-infected CD46+/IFNγ-KO pups at 3 and 7 dpi compared to uninfected mice (Fig. 4d) and CD46+ pups at the same time points (Fig. 4a). However, the %BrdU+ cells within the nestin+ cell population showed no significant difference between control and MV-infected pups in both genotypes (Fig. 4b, e), suggesting that the loss of nestin+ cells in CD46+/IFNγ-KO mice (Fig. 4e) is not due to impaired proliferation of the NSPC pool.

To evaluate whether neuronal differentiation was affected, we quantified the percentage of BrdU+ cells within the early neuronal CD24+ (Fig. 4c, f) and DCX+ populations (not shown). We were unable to detect BrdU-DCX double-positive cells by flow cytometry, suggesting that either the DCX+ cell population is too small in number to detect a small fraction of the cells that may have taken up BrdU (less than 3 % of cell isolate is DCX+; Fig. 2c, g) or that this population is more mature than the CD24+ population and that fewer DCX+ cells are proliferating at the time points examined. BrdU uptake was significantly reduced within the CD24+ population at 10 dpi in MV-infected CD46+ (Fig. 4c) and CD46+/IFNγ-KO mice (Fig. 4f), corresponding to when severe signs of illness and morbidity occur in this model.

These results suggest that IFNγ does not directly maintain the NSPC population through enhanced proliferation and that the loss of nestin+ cells in CD46+/IFNγ-KO mice is not due to impaired proliferation. Additionally, it is unlikely that the reduction in nestin+ cells is due to NSPCs being promoted to the neuronal lineage, as CD24+ and DCX+ populations are reduced and not increased, as would be expected with NSPCs adopting a neuronal cell fate.

### MV infection induces widespread apoptosis in brain tissue

**Fig. 5:**
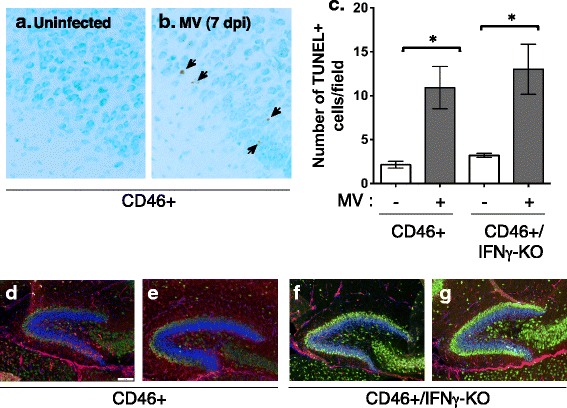
MV infection induces extensive apoptosis, but does not alter nestin localization. CD46+ and CD46+/IFNγ-KO brains were assayed by TUNEL (**a**–**c**) or immunohistochemistry for nestin and NeuN at 7 dpi (**d**–**g**). The average TUNEL+ cells per field (four fields per slice) were averaged from three different sagittal sections per mouse (**c**; *n* = 3 mice per condition). Significant increases in TUNEL+ cells were seen during infection in both genotypes, but no significant difference was observed between genotypes (**c**; *p < 0.01 significantly different uninfected versus MV-infected). Nestin (*red*) and NeuN (*green*) staining was observed in the hippocampus at 7 dpi in both CD46+ (**d**, **e**) and CD46+/IFNγ-KO (**f**, **g**) mice during infection (*scale bar* = 100 μm)

IFNγ may protect against toxic insults or induce apoptosis, depending on the type of neural cell [36, 38]. If IFNγ contributed to protection of NSPCs in the CD46+ model, then we would predict greater apoptosis in CD46+/IFNγ-KO mice during infection. To test whether IFNγ was associated with less apoptosis, we performed TUNEL stains on brain tissue from MV-infected mice (7 dpi). Significant TUNEL reactivity was found throughout the brains of MV-infected mice in comparison to uninfected controls (Fig. 5 a–c). No significant differences were found between CD46+ and CD46+/IFNγ-KO mice, suggesting that IFNγ does not reduce the number of cells undergoing apoptosis across the whole brain. We further examined brain tissue for staining of NeuN (mature neurons; green) and nestin (NSPCs, red) to assess if differences in neuronal health could be observed. Overall, we did not observe changes in the morphology of the hippocampal formation (Fig. 5 d–g) or cortex (not shown). These results demonstrate that although apoptosis is seen throughout the brain during infection, the level of cell death is not sufficient to cause marked changes in the structure of the brain.

### IFNγ preserves NSPC, but not neuronal, markers within the hippocampus during infection

Immunohistochemical staining for MV matrix and nucleocapsid proteins showed widespread MV-infection at 7 dpi (not shown) that was restricted to neuronal cell bodies and axonal processes. Although the infection was widespread, the hippocampus and surrounding area consistently displayed a “hot-spot” or focal area of MV-infected neurons at later dpi, compared to other brain regions, as has been noted previously [40]. As well, an increase in TUNEL+ apoptotic cells in the dentate gyrus of the hippocampus was reported at 7 dpi [50], suggesting that in addition to being a neurogenic region rich in NSPCs, the hippocampus is susceptible to the effects of and negatively impacted by MV infection. To evaluate effects on various cell types within the hippocampus, we attempted to isolate cells from neonatal hippocampal explants, but due to technical limitations we were unable to isolate enough cells for cell labeling and flow cytometry. Instead, we performed Western Blot analysis on neonatal hippocampal explants in order to evaluate changes in cell marker protein levels.

**Fig. 6:**
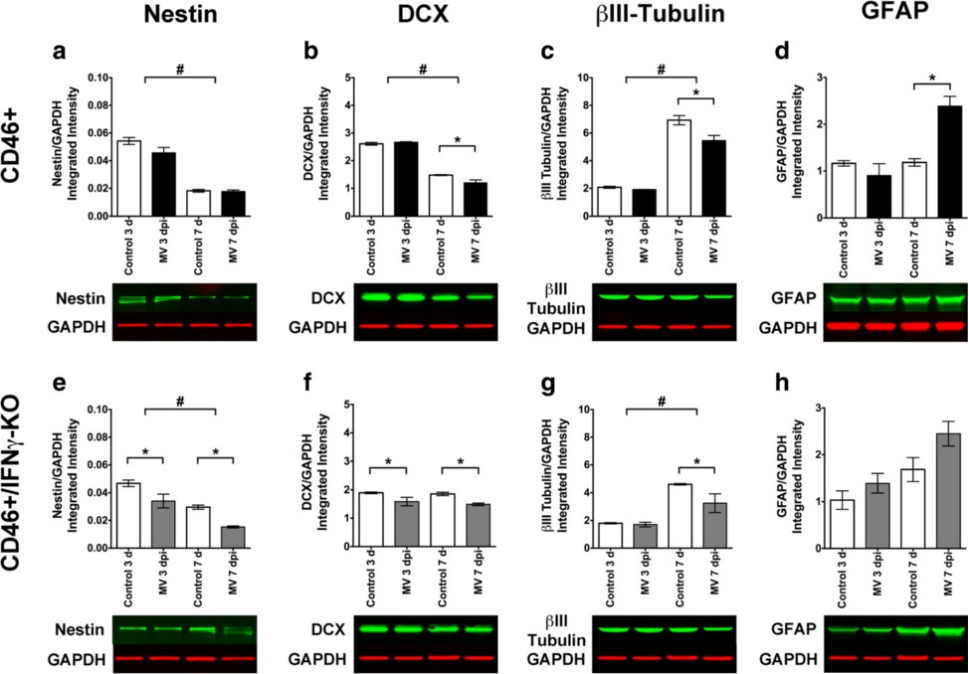
Impact of MV infection on expression of NSPC, neuronal, and glial markers in the hippocampus. Whole tissue lysates of hippocampal explants from CD46+ (**a**–**d**) and CD46+/IFNγ-KO (**e**–**h**) control and MV-infected mice were analyzed by Western Blots for Nestin (NSPC; **a**–**e**), DCX (early neuron; **b**, **f**), βIII-tubulin (mature neuron; **c**, **g**) and GFAP (astrocyte; **d**, **h**) proteins. Integrated intensity values for each band, which are proportional to the sum of the fluorescent signal (measured in pixels) enclosed by the region of interest, are normalized to GAPDH as a protein loading control. Changes in nestin and DCX hippocampal protein expression correspond with changes in cell number observed for whole-brain cell isolates. *p < 0.01 significantly different uninfected versus MV-infected; #*p* < 0.05 significantly different between dpi

**Fig. 7:**
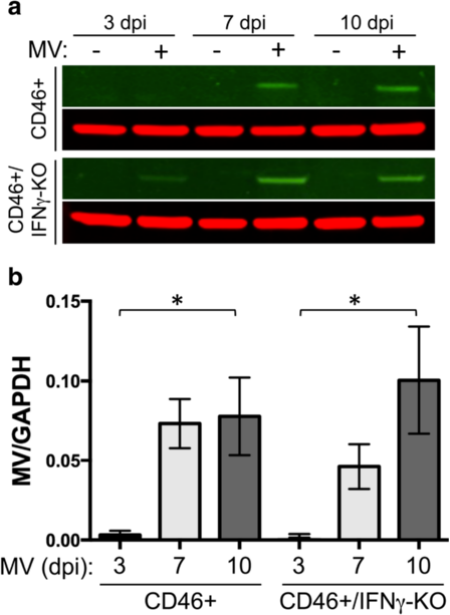
IFNγ does not reduce measles virus antigen in neonatal brain. **a**. Lysates of brain tissue from CD46+ and CD46+/IFNγ-KO neonates were analyzed by Western Blot for measles virus antigen. Control and MV-infected neonatal hippocampal lysates were collected at 3, 7, and 10 days post-infection (dpi) and probed with human polyclonal serum against MV and GAPDH as a loading control. **b.** Quantitation of MV levels normalized to GAPDH for each treatment condition in **a**. Statistical analysis was applied by two-way ANOVA (*n* = 4; *p < 0.01)

Changes in protein level in the hippocampus reflected changes in cell numbers that were measured by flow cytometry for NSPCs and immature neurons. MV-infected CD46+/IFNγ-KO explants displayed significantly lower nestin expression (Fig. 6e) compared to MV-infected CD46+ mice (Fig. 4a), which displayed no change with infection. Furthermore, there was a significant reduction in DCX protein at 7 dpi, not 3 dpi, in CD46+ pups (Fig. 6b), whereas DCX levels were reduced at all time points in CD46+/IFNγ-KO mice (Fig. 6f). βIII-tubulin levels were higher at 7 dpi compared to 3 dpi in both genotypes consistent with continued maturation of neurons; however, a significant decrease in βIII-tubulin was observed at 7 dpi in MV-infected explants, suggesting that the viral infection is detrimental to mature neurons, in addition to NSPCs (Nestin; Fig. 2a, e) and young neurons (DCX; Fig. 2b, f), in the hippocampus. The βIII-tubulin results contrast that observed for %NeuN+ cells quantified by flow cytometry (Fig. 2d, h), where no effect on mature neuron cell number was observed across the whole brain. GFAP levels were elevated significantly with infection in CD46+ mice, but not in CD46+/IFNγ-KO mice, in comparison to uninfected controls at 7 dpi, suggesting that localized reactive astrogliosis may be occurring in the hippocampus in the presence of IFNγ during infection (Fig. 6d, h). As a measure of viral load, we also quantified levels of MV protein using a polyclonal human antiserum (Fig. 7a). MV protein expression increased over the course of infection, with no significant difference in viral protein levels detected between genotypes (Fig. 7b). Thus, the differences we observed in levels of neural expression markers are not likely to be due to variances in viral load between the genotypes. Together, these results provide a secondary measure of cell protein content and validate the flow cytometry results within a neurogenic region of the brain that consistently carries a high viral load.

### STAT1 phosphorylation in the hippocampus occurs independently of IFNγ during MV infection

Since IFNγ affected neural cell marker expression in the hippocampus (Fig. 6) and the whole brain (Fig. 2; NSPC and neuron loss), we evaluated which IFN-signaling pathways are activated in the hippocampus during a viral infection. Type I interferons (IFNα and IFNβ) activate STAT1 and STAT2 proteins via phosphorylation and are required for early control of a viral infection. IFNγ, a Type II interferon, predominately activates STAT1, but can also activate STAT3 in neurons [56] and NSPCs [57]. In particular, IFNγ is required for long-term control of neurotropic viruses in CD46+ adult mice and other infection models [22, 58]. Thus, we analyzed hippocampal explants for expression of STAT1-3 and activation of the STAT proteins via phosphorylation.

**Fig. 8:**
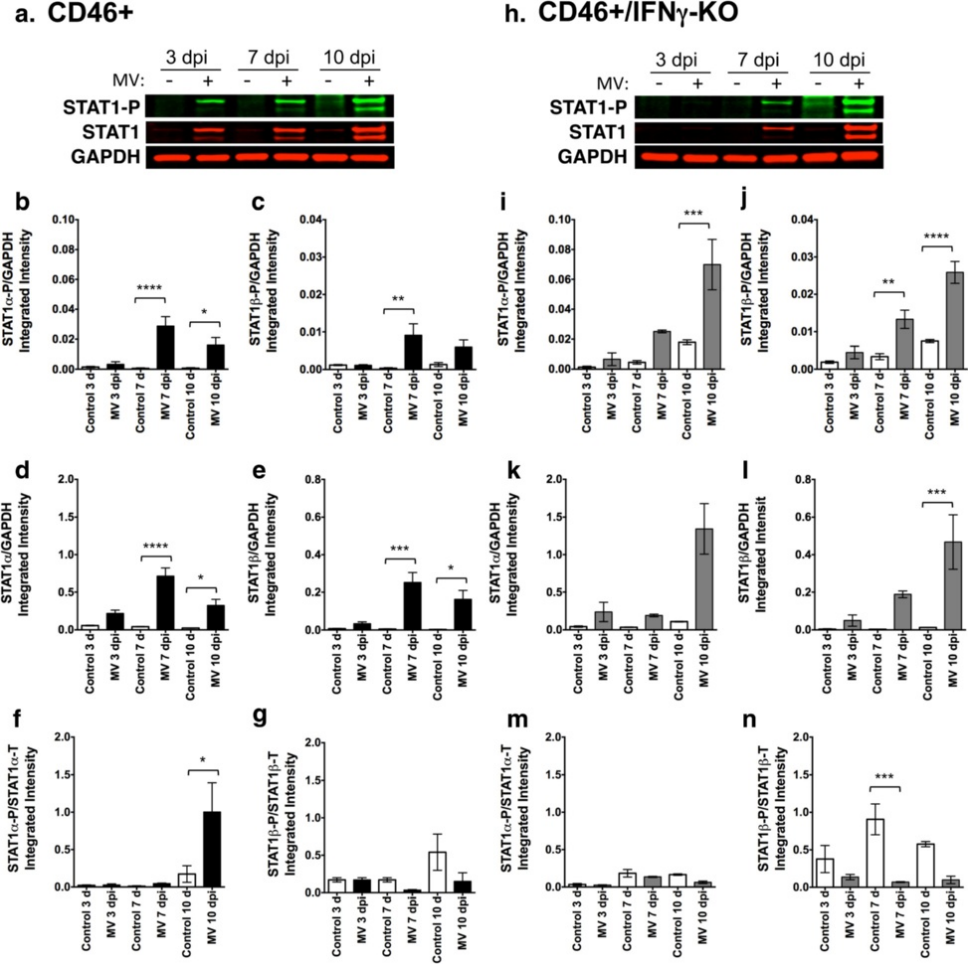
STAT1α and STAT1β isoforms in the hippocampus are phosphorylated in the absence of IFNγ during a viral infection. Lysates of hippocampal explants from CD46+ (**a**–**g**) and CD46+/IFNγ-KO (**h**–**n**) control and MV-infected mice were analyzed by western blot for phosphorylated (activated) STAT1 (**b**, **c**, **i**, **j**), and total STAT1 (**d**, **e**, **k**, **l**) proteins. GAPDH was used as a protein loading control. *Representative blots* are shown in **a** and **b**. Total levels of STAT1α (upper band; **d**, **k**) and STAT1β (lower band; **e**, **l**) were significantly increased in MV-infected hippocampal explants from CD46+ pups (**d**, **e**) at 7 and 10 dpi and in CD46+/IFNγ-KO pups (**k**, **l**) at 10 dpi. Phosphorylation of STAT1α (STAT1α-P; B, I) increased significantly in CD46+ explants at 7 and 10 dpi (**b**) and CD46+/IFNγ-KO explants at 10 dpi (I). Phosphorylation of STAT1β (**c**, **j**) was increased in CD46+ explants at 7 dpi only (**c**) and in CD46+/IFNγ-KO explants and 7 and 10 dpi (**j**). Protein ratios of STAT1α-P/STAT1α showed increased activation of phosphorylation of STAT1α-P at 10 dpi in CD46+ mice (**f**), but no activation in CD46+/IFNγ-KO mice (**m**). The protein ratios of STAT1β-P/STAT1β showed decreased activation of phosphorylation during infection in CD46+/IFNγ-KO mice (**n**), but not in CD46+ mice (**g**). Statistical analysis was applied by one-way ANOVA with multiple comparisons. (**p < 0.01, ***p < 0.001, ****p < 0.0001 significantly different uninfected versus MV-infected; *n* = 4)

**Fig. 9:**
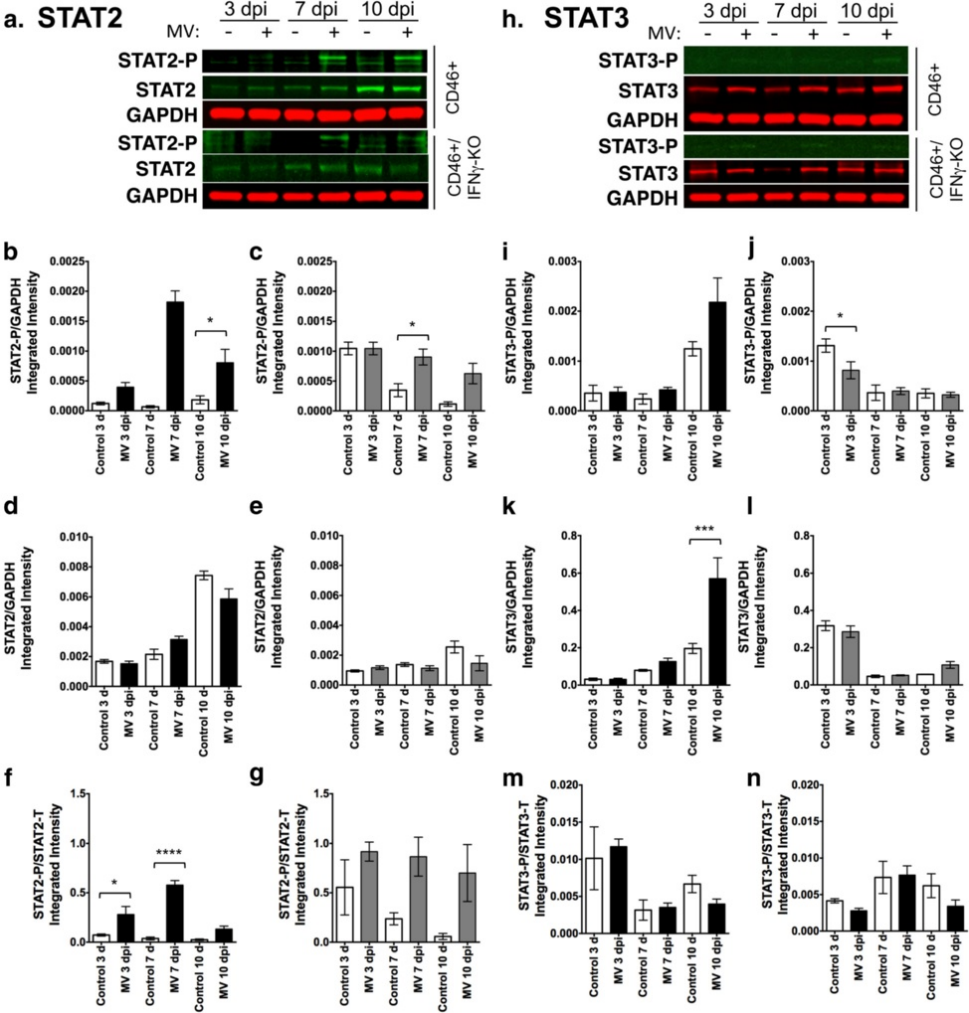
STAT2, but not STAT3, is activated during MV infection in the neonatal hippocampus. Lysates of hippocampal explants from CD46+ (black bars; **b**, **d**, **f**, **I**, **k**, **m**) and CD46+/IFNγ-KO (grey bars; **c**, **e**, **g**, **j**, **l**, **n**) control and MV-infected mice were analyzed by western blot for phosphorylated (activated) STAT2 (STAT2-P; **b**, **c**), total STAT2 (**d**, **e**), STAT2-P/STAT2 (**f**, **g**), STAT3-P (**i**, **j**), total STAT3 (**k**, **l**), and STAT3-P/STAT3 (**m**, **n**). GAPDH was used as a protein loading control. Representative blots are shown for STAT2 (**a**) and STAT3 (**h**). STAT2-P levels were significantly increased in MV-infected hippocampal explants in CD46+ (**b**) and CD46+/IFNγ-KO (**c**) pups at 7 dpi, though STAT2 phosphorylation was sustained to 10 dpi in CD46+ explants. Protein ratios of STAT2-P/STAT2 showed significantly increased activation of phosphorylation in CD46+ mice at 3 and 7 dpi (**f**). STAT3 phosphorylation was not significantly induced under any condition (**g**, **h**, **m**, **n**). Levels of total STAT3 protein were increased with infection at 10 dpi in CD46+ explants (**i**), but not in CD46+/IFNγ-KO explants (**j**). Statistical analysis was applied by one-way ANOVA with multiple comparisons (*p < 0.05, ***p < 0.001, ****p < 0.0001 significantly different uninfected versus MV-infected; *n* = 4)

Hippocampal tissue lysates were analyzed for total (STAT) and phosphorylated (STAT-P) forms of STAT proteins that are activated by IFNα/β (STAT1, STAT2) and IFNγ (STAT1, STAT3) (Figs. 8 and 9). STAT1 is expressed as two isoforms; STAT1α, which exhibits DNA binding activity, and STAT1β, which lacks a DNA binding domain and acts as negative regulator of STAT1α. Each isoform of STAT1 was assayed independently in Fig. 8. Total STAT1 expression and STAT1 phosphorylation was unchanged at 3 dpi in both genotypes. In CD46+ explants, STAT1α was significantly activated via phosphorylation above untreated controls at 7 and 10 dpi (Fig. 8b), whereas STAT1β was significantly activated at 7 dpi (Fig. 8c) when normalized to GAPDH. CD46+/IFNγ-KO explants also demonstrated activation of STAT1α and STAT1β, with significant activation of both isoforms at 10 dpi (Fig. 8g, h). Total levels of STAT1 expression increased at later time points post-infection in both genotypes. CD46+ explants showed significantly more expression of total STAT1α and β at both 7 and 10 dpi (Fig. 8d, e), while significantly higher expression of total STAT1 was only observed at 10 dpi in the absence of IFNγ (Fig. 8i, j). These results demonstrate that phosphorylation of STAT1 occurs in hippocampal tissue in the absence of IFNγ during infection, potentially through expression of Type I IFNs. Protein ratios of STAT1-P/STAT1 revealed distinct regulation of STAT1 phosphorylation between genotypes that was isoform dependent (Fig. 8f, g, m, n). CD46+ mice showed a significant increase in the STAT1α-P/STAT1α ratio at 10 dpi, which was absent at all time points in the absence of IFNγ. Moreover, STAT1β-P/STAT1β ratios declined during infection in CD46+/IFNγ-KO explants, but not CD46+ explants. These data suggest IFNγ drives an increase in phosphorylation of the available STAT1α in the cell, although increased expression of both STAT1 isoforms occurs independently of IFNγ.

We further explored the activation of other STAT molecules (STAT2 and STAT3) that have been implicated as alternative signaling pathways downstream of IFNγ [56, 59] (Fig. 9). STAT2 phosphorylation is induced in CD46+ explants at 7 and 10 dpi by MV infection (Fig. 9b), whereas significant phosphorylation of STAT2 was only seen at 7 dpi in CD46+/IFNγ-KO explants (Fig. 9c) when normalized to GAPDH. Total expression of STAT2 did not change significantly with infection in either genotype (Fig. 9d, e). However, total STAT2 levels increased independently of infection over time in CD46+ explants (Fig. 9d). Protein ratios of STAT2-P/STAT2 revealed increased activation of phosphorylation of STAT2 in CD46+ mice, with a trend toward increased regulation in CD46+/IFNγ-KO mice (Fig. 9f, g). In contrast, STAT3 activation did not increase with infection (Fig. 9i, j, m, n). Total STAT3 expression increased at 10 dpi in CD46+ explants, which was not observed in the absence of IFNγ (Fig. 9i, j), suggesting either IFNγ or other inflammatory cytokines that are induced by IFNγ (e.g., IL-6) may lead to greater STAT3 expression [60]. Together, these results demonstrate that activation of STAT signaling molecules does not differ significantly early in infection (3 dpi), but that STAT1 and STAT2 phosphorylation occurs later infection in CD46+ and CD46+/IFNγ-KO explants. Thus, phosphorylation of STAT1 in the brain parenchyma may be influenced by cytokines other than IFNγ during MV infection.

## Discussion

This is the first study to directly examine the bystander effects of the anti-viral immune response on NSPC function in a model with neuron-restricted viral infection. Restricted NSPC proliferation and cell loss have been reported for various neurotropic viruses, but NSPCs were targets for the virus in many of these models [15, 17, 61]. Thus, the contributions from either the virus or the immune response to alterations in the NSPC pool are still unclear. In the CD46+ mouse model, NSPCs are spared from MV-infection. We found that NSPCs were protected by IFNγ in MV-infected pups, whereas significant immature neuron loss was observed in both CD46+ and CD46+/IFNγ-KO pups. Mature neuron and glial cell numbers were unaltered. The proliferative index of the NSPC populations was unaffected while neurogenesis only declined at 10 dpi, suggesting that cell loss in both NSPC and early neuron populations is likely due to cell death and not due to changes in cell fate.

In the absence of IFNγ, the NSPC pool declined significantly at 7 and 10 dpi, with a trend toward lower NSPC numbers at 3 dpi (Fig. 2). The earliest time point that we examined (3 dpi) is associated with a negligible number of T-cells in the brain but with notable natural killer cell infiltration (P. Ganesan, unpublished observations). It is possible that IFNγ production by T-cells is required to afford protection to the NSPC pool at later time points in infection. We also noted a lack of an effect on NSPC proliferation (Fig. 4), which we speculate is due to NSPCs remaining uninfected in our model. NSPCs infected with murine cytomegalovirus (MCMV) [17, 62], lymphocytic choriomeningitis virus (LCMV) [63], Japanese encephalitis virus (JEV) [61], and herpes simplex virus, type 1 (HSV-1) [15] display reduced proliferation that is associated with viral infection and replication in NSPCs. Instead, in our model, we suspect that the loss of uninfected NSPCs is due to cell death in the CD46+/IFNγ-KO pups. If NSPCs were undergoing a change in cell fate, we would have expected an increase in the number of neurons or glia, but instead we observed a reduced and unchanged cell number, respectively (Figs. 2 and 3), suggesting that the NSPCs are not being lost due to commitment to other cell types. Since direct viral infection cannot account for NSPC loss in our model system, one possibility is that the cytokine milieu that is released in the absence of IFNγ is ultimately detrimental to the cells. A similar phenomenon has been characterized in models of antigen-induced arthritis, where IFNγ-KO mice suffer greater disease severity due to overexpression of IL-17 [64]. Additionally, an outstanding question that remains is how the presence of IFNγ leads to protection of the NSPC pool in CD46+ neonates. IFNγ treatment of NSPCs in vitro leads to decreased proliferation in the absence of other cytokines [65], which implies that IFNγ does not afford protection to NSPCs by increasing proliferation in our model. Defining the anti-viral cytokine profile in CD46+ and CD46+/IFNγ-KO pups may provide insight into the milieu that is required to maintain the NSPC pool during infection.

In CD46+ pups, young neurons are more susceptible to cell loss than mature neurons. Newly committed neuronal cells were lost at all time points post-infection in CD46+/IFNγ-KO pups and at 7 and 10 dpi in CD46+ pups (Fig. 2). The production of new neurons, as measured by BrdU incorporation in CD24+ cells, declined only at 10 dpi in both genotypes. Thus, a decrease in neurogenesis cannot account for the loss of early neurons at other time point during infection (3 and 7 dpi, Fig. 4). One possibility is that early neurons are infected by MV as they fully differentiate, since the cells will begin to express NSE, and thus the human CD46 transgene, as MAP2 and βIII tubulin are induced [66]. In support of this possibility, NSPCs infected with Borna disease virus (BDV) and human immunodeficiency virus-1 (HIV-1) are unaffected by the viral infection. However, once cells are committed to the neuronal lineage and enter a post-mitotic state, apoptotic cell loss is observed [16, 67]. This led us to also consider whether the subset of immature neurons lost during infection were actually MV-infected. We attempted to co-label our neural cells for MV proteins (not shown) to determine if CD24+ and DCX+ cells were MV-infected. Unfortunately, we were unable to successfully label our cell isolates for MV proteins by flow cytometry. Regardless, neuronal death is a common pathological outcome of viral infection [14]. It is likely that the reduction in neuronal cell number we observed was due to cell death and not due to NSPCs adopting a non-neuronal (glial) fate. Furthermore, adult CD46+ mice exhibit only limited apoptosis as a result of MV infection, which corresponds with successful viral control and survival, whereas a higher number TUNEL+ cells and death occurs in MV-infected CD46+ neonates at 7 dpi and beyond (Fig. 5; [19, 50]). Though adult CD46+ mice are capable of mediating non-cytolytic clearance of MV from CNS neurons, neonatal CD46+ mice cannot mediate non-cytolytic clearance, as evidenced by persistent viral spread and widespread apoptosis in the brain parenchyma, despite T-cell infiltration [19, 49, 50]. Differences in non-cytolytic responses from neonatal and adult T-cells are poorly understood. However, our findings imply that the neonatal immune response not only fails to control MV, but may also contribute to a loss of early neurons during MV infection. In support of this hypothesis, in vitro results demonstrate that embryonic CD46+ neurons are not killed by MV infection directly [68], suggesting that other factors contribute to loss of early neurons in vivo.

Western Blot analysis on hippocampal explants displayed changes in protein expression levels that corresponded with effects on cell number for nestin and DCX, but not for βIII-tubulin or GFAP (Fig. 6). The percentage of mature NeuN+ cells was not significantly different in MV-infected explants compared to controls by flow cytometry (Fig. 2), but a significant reduction in βIII-tubulin expression was observed in MV-infected samples at 7 dpi. It is possible that the virus exerted regional effects on neuron survival and that hippocampal neurons were more susceptible to infection at 7 dpi compared to other neurons elsewhere in the brain. Another possibility is that the effects on hippocampal neurons may not have been detectable in whole-brain isolates where neurons from the hippocampus are a small fraction of the total number of neurons isolated. Alternatively, Zimmer and colleagues observed that βIII-tubulin expression is downregulated at the transcriptional level by low doses of toxins, without frank cell death [69]. Thus, loss of βIII-tubulin may be indicative of stress on the hippocampal neurons in our model, but the stressor may not be sufficient to cause cell death. For GFAP staining, flow cytometry did not reveal significant changes in the number of GFAP+ cells (Fig. 3c, g) or in the intensity of the GFAP signal in whole-brain isolates (Fig. 3d, h). However, at 7 and 10 dpi, a trend toward an increased number of GFAP+ cells and greater GFAP intensity was observed, though this change was not significant. When we examined GFAP levels by Western Blot analysis in hippocampal explants, there was a increase in GFAP expression at 7 dpi in MV-infected CD46+ mice compared to uninfected CD46+ mice (Fig. 6d) which was not observed in the absence of IFNγ. A possible explanation for the discrepancy in our data is that the hippocampus supports a high viral load in the CD46+ model. Therefore, the astrocytes in the hippocampus would be more likely to respond consistently to infected neurons, thus making changes in GFAP more readily detectable in this region.

Having observed that IFNγ is protective for NSPCs (Fig. 1), we attempted to characterize which IFNγ-related signaling pathways are active in infected brain tissue. We also considered that Type I interferons (IFNα and IFNβ) may be functioning as protective factors in the CD46+ pups. These IFNs also signal through STAT signaling pathways that are known to function in various CNS pathologies [70]. Furthermore, STAT1 signaling is important for viral control in adult CD46+ mice before 6 dpi, when the innate immune response is active [58]. We observed increased phosphorylation of STAT1 and STAT2, but not STAT3, in the hippocampi of MV-infected pups (Figs. 8 and 9). We had predicted that STAT1 signaling would be more pronounced in CD46+ pups, since the CD46+/IFNγ-KO pups would not have IFNγ available to activate STAT1. STAT1 phosphorylation occurred in the absence of IFNγ during MV infection, with CD46+/IFNγ-KO showing pronounced phosphorylation of both STAT1 isoforms when normalized to GAPDH versus uninfected controls (Fig. 8i, j). However, protein ratios for STAT1α-P/STAT1α showed that only CD46+ mice increased regulation of phosphorylation during infection (Fig. 8f, m). Moreover, STAT1β-P/STAT1β ratios revealed decreased regulation of phosphorylation in the absence of IFNγ (Fig. 8n). These results suggest that IFNγ drives both increased phosphorylation of STAT1α, which is the transcriptionally active form of STAT1, during infection. Furthermore, although the relative level of phosphorylation of the dominant-negative isoform of STAT1 (STAT1β) increased in both genotypes (Fig. 8c, j), the regulation of STAT1β-P phosphorylation declined in the absence of IFNγ. One possibility is that other cytokines (e.g., IFNα/β) contribute to STAT1 phosphorylation during the anti-MV immune response, thus causing distinct patterns of STAT1 phosphorylation between genotypes. In CD46+ pups, STAT1 phosphorylation peaked at 7 dpi and then declined by 10 dpi (Fig. 8b–e). STAT2 activation also demonstrated a similar pattern to STAT1 (Fig. 9b), suggesting that a subset of signaling events was declining toward the end of the time course. Most of the CD46+ pups succumb to the infection between 9–12 dpi regardless of IFNγ expression [49], and it is unclear why the CD46+ pups would demonstrate a decline in inflammatory signaling pathways when the virus is still present. However, future studies will examine the cytokine expression profile over the course of infection in CD46+ pups, which may provide an explanation as to the reduction in STAT1 and STAT2 signaling by 10 dpi.

Viral infections in the brain induce microglial and astrocytic activation, in addition to infiltration of peripheral immune cells. Widespread F4/80+ activated microglial staining was reported in MV-infected NSE-CD46+ pups at 7 dpi [50], leaving open the possibility that the effects we observed on NSPCs and immature neurons were due to indirect effects on IFNγ-responsive microglia during infection. Activated microglia release pro-inflammatory factors that can positively or negatively impact neuronal function [71–73]. For example, IFNγ-activated microglia were shown to promote neurogenesis from NSPCs and to promote neuronal survival in vitro [74, 75]. In our study, the loss of IFNγ may have resulted in reduced microglial activation that compromised NSPC number in CD46+/IFNγ-KO mice but not CD46+ mice, due to a loss of protection or a loss of trophic support. We also observed a loss of immature neurons in both CD46+ and CD46+/IFNγ-KO pups, suggesting that IFNγ does not protect this population (Fig. 1). Therefore, another possibility is that microglial activation is detrimental to early neuron survival, independent of IFNγ. Microglial activation has been shown to cause neuronal apoptosis in the cortex, cerebellum, and hippocampus of BDV-infected neonatal rats in vivo [76, 77]. In our model, subpopulations of microglia may be more or less reliant on IFNγ for activation and in our model this may have contributed to protection over NSPCs, but not early neurons, respectively.

Our studies are focused on the role of IFNγ in viral pathogenesis in the CNS. However, IFNγ is also expressed in many neurodegenerative disease states [78–80] where alterations in NSPC function are also observed. Though the pathology of these disease states varies, an understanding of the contribution of key cytokines, as well as combinations of multiple inflammatory mediators, will be necessary to fully understand the impact of the immune response on NSPC activity and survival.

## Conclusions

This is the first study to demonstrate that IFNγ protects NSPCs during a CNS viral infection. A transgenic mouse model of neuron-restricted MV infection was used to test the effects of the inflammatory environment created by the anti-viral immune response on NSPCs. Our results are the first to directly show that anti-viral immunity imposes bystander effects that compromise NSPC cell number, in the absence of IFNγ. It is still unclear what component of the immune response is damaging to NSPC function. Recent work by Hu and colleagues indicated that activated CD8+ T-cells, but CD4+ T-cells, reduced proliferation of MHC-matched NSPCs through the release of IFNγ in vitro [81]. While we did not observe a decline in NSPC proliferation in our in vivo model, these results highlight the importance of considering the roles of specific immune cells in affecting NSPC function. It will be interesting to identify the immune cell subsets that are present during the 3–10 dpi window in order to clarify which immune cell population compromises NSPC and early neuron cell number. Overall, this study implicates IFNγ as one cytokine involved in preserving NSPCs during viral infections and other inflammatory conditions.

## Additional file

Additional file 1:
**Figure S1.** Representative flow cytometry plots for neural cell identification. Brain homogenates from neonatal mice were analyzed via flow cytometry. Representative plots for each IgG isotype control and the respective neural cell antibody are shown. Top row: Forward/side scatter and 7-AAD negative (−) gates were applied to all samples. 2nd row: Markers for neural stem cells (nestin) and early neuronal markers (doublecortin, DCX). 3rd row: Markers for early neurons (CD24) and for mature neurons (NeuN). 4th Row: Markers for early glial progenitors (A2B5) and mature astrocytes (GFAP). (PDF 504 kb)

## Abbreviations

ANOVA: Analysis of variance
ATCC: American Type Tissue Collection
BDV: Borna disease virus
BrdU: 5-bromo-2′-deoxyuridine
CNS: Central nervous system
Dpi: Day post-infection
DCX: Doublecortin
GAPDH: Glyceraldehyde 3-phosphate dehydrogenase
GFAP: Glial fibrillary acidic protein
HIV-1: Human immunodeficiency virus-1
KO: Knockout
HSV-1: Herpes simplex virus, type 1
IFN: Interferon
IFNα: Interferon-alpha
IFNβ: Interferon-beta
IFNγ: Interferon-gamma
JEV: Japanese encephalitis virus
LCMV: Lymphocytic choriomeningitis virus
MAP2: Microtubule associated protein 2
MFI: Mean fluorescent intensity
MV: Measles virus
MCMV: Murine cytomegalovirus
MHC: Major histocompatibility complex
NSE: Neuron-specific enolase
NSPC: Neural stem/precursor cell
P: Postnatal day
PBS/tw: Phosphate-buffered saline and Tween-20
PFU: Plaque forming unit
SE: Standard error
STAT: Signaling transducer and activator of transcription
STAT-P: Phosphorylated signaling transducer and activator of transcription
TUNEL: Terminal deoxynucleotidyl transferase dUTP nick end labeling
