# Supplementary material for: Interferon gamma protects neonatal neural stem/progenitor cells during measles virus infection of the brain
Source: J Neuroinflammation. 2016 May 13;13:107. doi: 10.1186/s12974-016-0571-1 (PMC4867982; doi:10.1186/s12974-016-0571-1)
Supplement: Additional file 1: — Figure S1. Representative flow cytometry plots for neural cell identification. Brain homogenates from neonatal mice were analyzed via flow cytometry. Representative plots for each IgG isotype control and the respective neural cell antibody are shown. Top row: Forward/side scatter and 7-AAD negative (−) gates were applied to all samples. 2nd row: Markers for neural stem cells (nestin) and early neuronal markers (doublecortin, DCX). 3rd row: Markers for early neurons (CD24) and for mature neurons (NeuN). 4th Row: Markers for early glial progenitors (A2B5) and mature astrocytes (GFAP). (PDF 504 kb) [file 12974_2016_571_MOESM1_ESM.pdf]

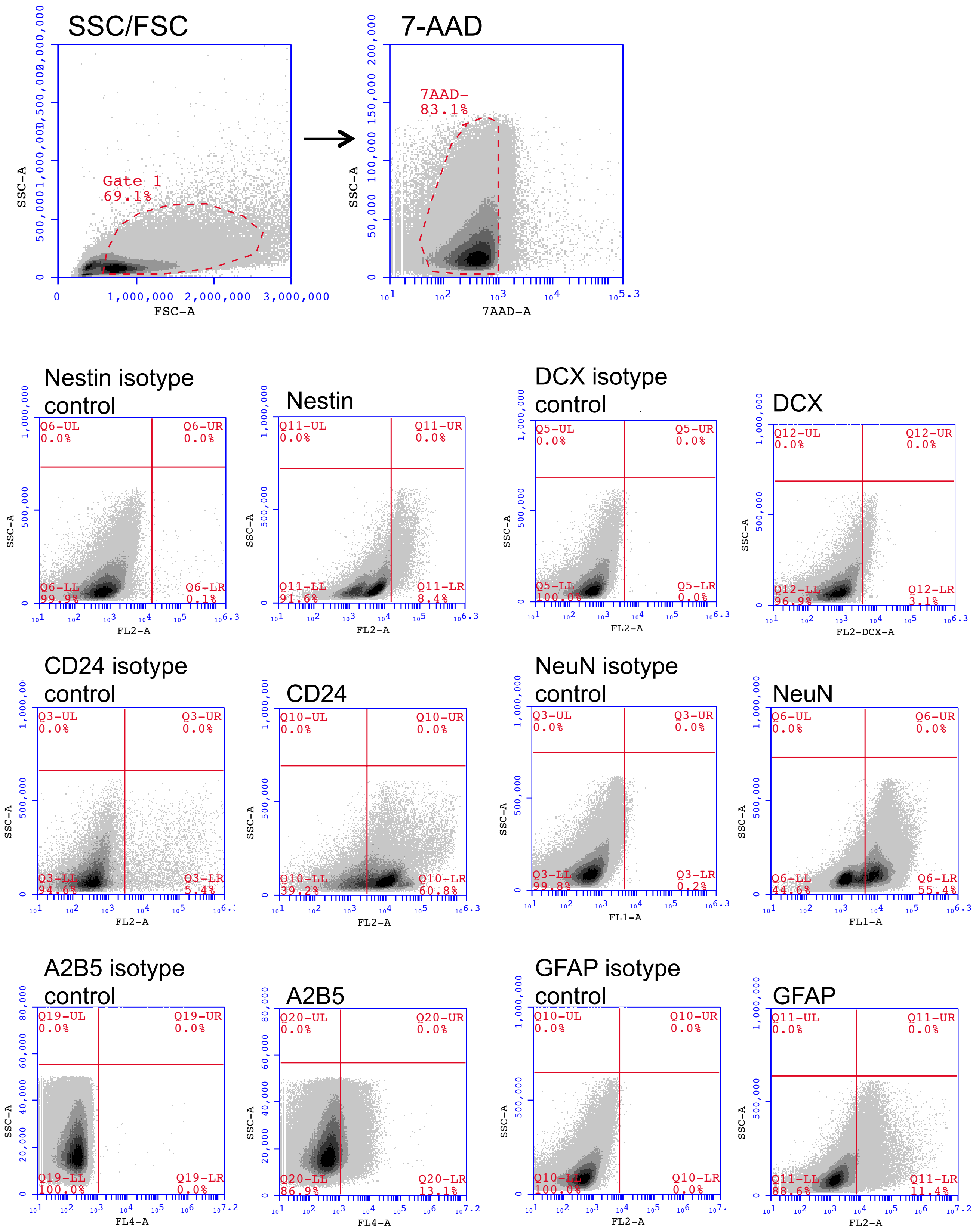

Supplemental Figure 1. Representative flow cytometry plots for neural cell identification. Brain homogenates from neonatal mice were analyzed via flow cytometry. Representative plots for each IgG isotype control and the respective neural cell antibody are shown. Top row: Forward/side scatter and 7-AAD negative (-) gates were applied to all samples. 2nd row: Markers for neural stem cells (nestin) and early neuronal markers (doublecortin, DCX). 3rd row: Markers for early neurons (CD24) and for mature neurons (NeuN). 4th Row: Markers for early glial progenitors (A2B5) and mature astrocytes (GFAP).
